# Assessing silvopasture management as a strategy to reduce fuel loads and mitigate wildfire risk

**DOI:** 10.1038/s41598-024-56104-3

**Published:** 2024-03-12

**Authors:** Mark Batcheler, Matthew M. Smith, Mark E. Swanson, Marcia Ostrom, Lynne Carpenter-Boggs

**Affiliations:** 1 Corvallis Forestry Sciences Laboratory, USDA National Agroforestry Center, 3200 Southwest Jefferson Way, Corvallis, OR 97331 USA; 2 USDA National Agroforestry Center, 1945 North 38Th Street, Lincoln, NE 68583 USA; 3 School of the Environment, Washington State University, P.O. Box 64610, Pullman, WA 99164 USA; 4 School of the Environment, Washington State University, 1100 North Western Avenue, Wenatchee, WA 98801 USA; 5 Department of Crops and Soils, Washington State University, Pullman, WA 99164 USA

**Keywords:** Agroecology, Forest ecology

## Abstract

Managing private forests for wildfire resilience is challenging due to conflicting social, economic, and ecological decisions that may result in an increase of surface fuel loads leading to greater fire risk. Due to fire suppression and a changing climate, land managers in fire-prone regions face an increasing threat of high severity fires. Thus, land managers need fuel treatment options that match their forest types and management objectives. One potential option for producers that graze livestock is silvopasture management, where livestock, forages, and overstory vegetation are carefully managed for co-benefits on the same unit of land. This study compared forest composition and structure, fuel types, and vegetative biomass between silvopasture and non-grazed managed forests in Washington, U.S. We show that silvopasture management results in reductions in grass biomass, litter, and duff depth when compared to non-grazed managed forest. These findings point to the integrated nature of silvopasture, where management of overstory composition and structure, understory vegetation, and grazing can reduce fuel loads and potential wildfire risk.

## Introduction

Wildfire is an integral ecological disturbance that can benefit the integrity of western U.S. forests, particularly in dry pine forests^1^. However, fire frequency has deviated from historic fire regimes, threatening human communities due to increased development in the wildland urban interface^2,3^. The drivers of these wildfires are exacerbated by a combination of interlinked factors, including climate change, historic fire suppression, unrestricted grazing, and forest management practices, resulting in shifts in forest structure and species composition^3–5^. Forest management aimed at mitigating wildfires is driven by complex social and ecological factors, requiring forest management strategies to transition forest ecosystems to ones that are more fire adapted and resilient, while still maintaining ecological complexity^6^. Wildfire management is a concern for privately owned lands in the western U.S. as indicated by a recent study that determined that 60% of fires occurring between 1992 and 2017 began on these lands^7^. Further, human-caused climate change increases forest fire activity which contributes to large losses of carbon to the atmosphere compounding the issue^8^.

Addressing fire management on privately owned forests has an inherent complexity due to private landowners’ diverse and individualized management objectives. Well-established methods to reduce surface fuel loads may include prescribed fire^9–11^, thinning to promote structural heterogeneity^10,12^, and targeted grazing. Of these treatments, there is a rise in the use and analysis of targeted grazing as a viable means of reducing risk of wildfire^13–16^. Targeted grazing is defined as the application of seasonal livestock for a set duration and intensity to accomplish pre-determined vegetation or landscape goals^17^. This is not to be confused with continuous grazing in which livestock have unrestricted access to a unit of land for a set duration (also known as continuous stocking)^18^. Continuous grazing has had complex impacts on forest health, with several studies finding that the practice increased wildfire risk^19,20^. In contrast, targeted grazing has been successfully used to reduce surface fuel loads in wooded areas^21,22^ and grass/shrublands^23–25^. However, treating surface fuel loads through use of targeted grazing is not often coupled with overstory management, representing a possible opportunity to further reduce fuels.

Silvopasture, defined as the intentional integration of trees, forage, and livestock on the same unit of land^26^, differs from forest and targeted grazing in that all three system components are carefully managed to increase beneficial interactions. When compared to other forest grazing practices, silvopastures often have fewer trees per hectare, greater spacing between trees, and tree limbs are often pruned to the first sawlog^26^. Livestock are managed using rotational grazing, which allows for recurring periods of grazing and rest through the use of multiple paddocks^26,27^. Understory vegetation may be introduced or modified in order maximize forage production^27^. Because of the more deliberate and systems approach to management, silvopastures are capable of improving ecosystem services, such as carbon storage, nutrient cycling, water infiltration rates, and improved biodiversity^26^.

In several Mediterranean countries, research has been conducted on silvopasture as a means of reducing surface fuel loads^28^ and wildfire occurrence through the simultaneous management of the overstory, livestock, and understory vegetation^29–31^. The reduced density and increased spatial variability of the overstory, combined with other silvopasture management practices, has the potential of reducing fire frequency when compared to other land uses^32^. For livestock, use of rotational grazing in a silvopasture has been found to effectively remove understory biomass, thus reducing herbaceous surface fuel loads^28,33^. Livestock in silvopastures have also been found to reduce the volume of litter and duff by incorporating that biomass into the soil via trampling^34^. In terms of forages, pastoral improvements in the form of fertilization and overseeding of more palatable species have been found to reduce fuel loads in silvopastures due to more complete forage consumption by livestock^35^. Together, these management practices have shown the effectiveness of silvopasture at reducing fuel loads in the Mediterranean.

Outside of the Mediterranean region, few studies have assessed the efficacy of fuels reduction from silvopasture management. Given the increase in catastrophic wildfires that result in loss of life, natural resources, and property, research is needed to understand whether silvopasture management can be used as an effective tool for fuels mitigation in other regions and climates. Additionally, limited research has been conducted on whether the use of silvopasture can affect the long-term trajectory of ecosystems prone to wildfire. The objective of this research is to investigate the effects of silvopasture management on fuel loading, overstory and understory composition and structure, and shrub and herbaceous biomass. Results from this research fill a critical knowledge gap on whether silvopasture can be used as an effective fuels management tool for livestock producers in Washington State. Because of the prescriptive nature of silvopasture management, these results may be applicable to other regions with dryland forests.

## Methods

### Site description and management

**Figure 1:**
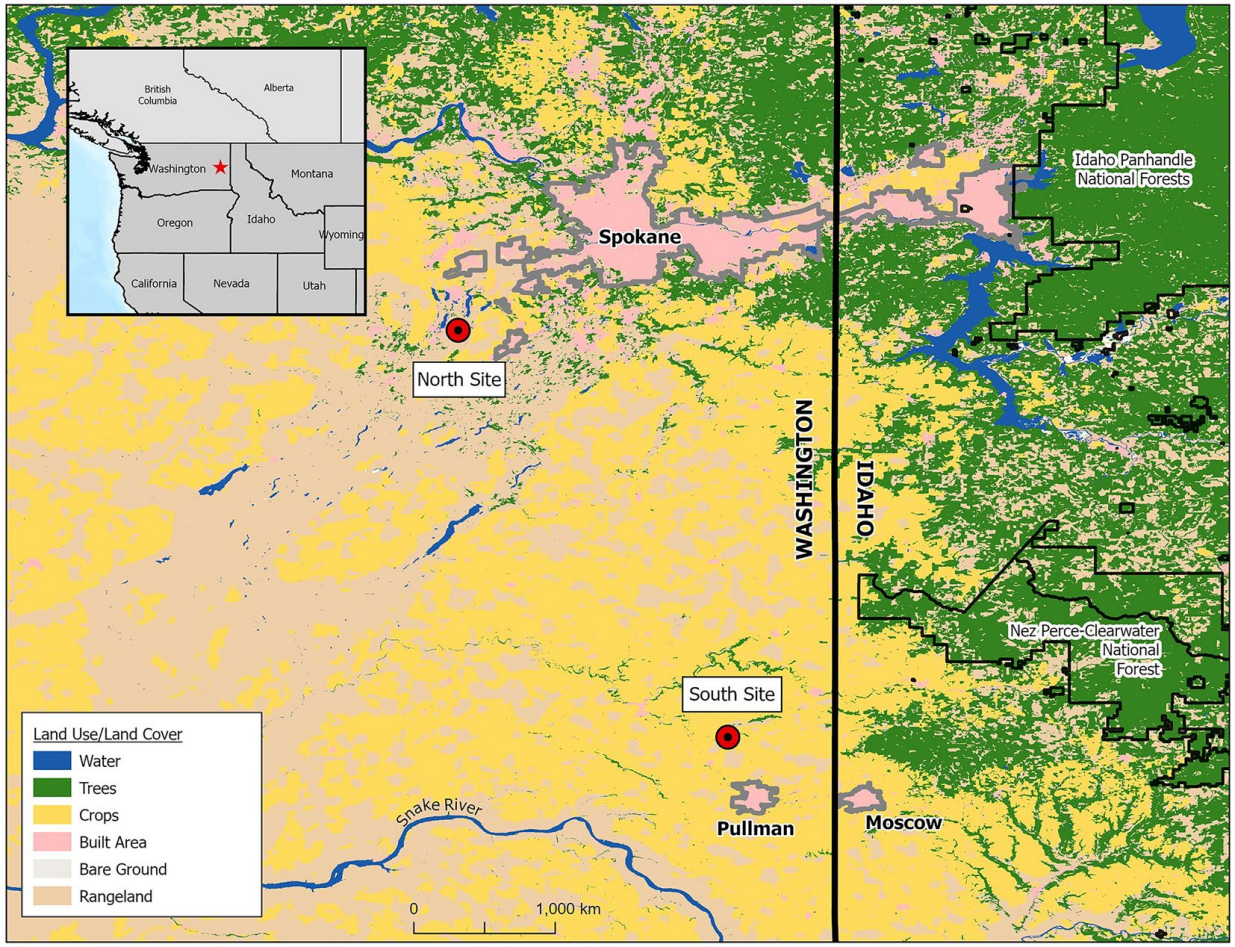
Research sites in Washington State. This map was generated using ArcGIS Pro 3.1.

This research was conducted on non-industrial privately owned forests in eastern Washington, U.S. (Fig. 1). Study sites were selected based on similar eco-physiography (topography, elevation, parent material, precipitation, and temperature regimes). Sites are characterized by the Köppen climate type represented by a warm-summer Mediterranean (Csb) and the sites are limited to dryland forest habitats in the Interior Columbia Basin. The northern site is in Cheney, Washington and has Northstar-Rockly complex silt loam soils. The southern site is located in Albion, Washington and is comprised of Gwin-Linville complex and Larkin silt loams. Site variables are presented in Table 1

**Table 1:** Summary of silvopasture and non-grazed managed forest site variables.

Location	Management system	Aspect (mean)	Slope (mean)	MAP (mm)	MAT (°C)	Elevation above sea level (m)	Management goals
Cheney	Silvopasture	E	3.0	459.5	8.2	711.2	Livestock production, fuels management, firewood
Cheney	Non-grazed managed forest	W–SW	3.2	459.5	8.2	767.4	Fuels management, firewood
Albion	Silvopasture	W–NW	19.6	544.2	8.8	714.6	Livestock production, fuels management
Albion	Non-grazed managed forest	E–SE	14.0	544.2	8.8	735.8	Timber, firewood, fuels management

Mean annual precipitation (MAP) and mean annual temperature (MAT) data are from the PRISM Climate Group^36^.

.

**Figure 2:**
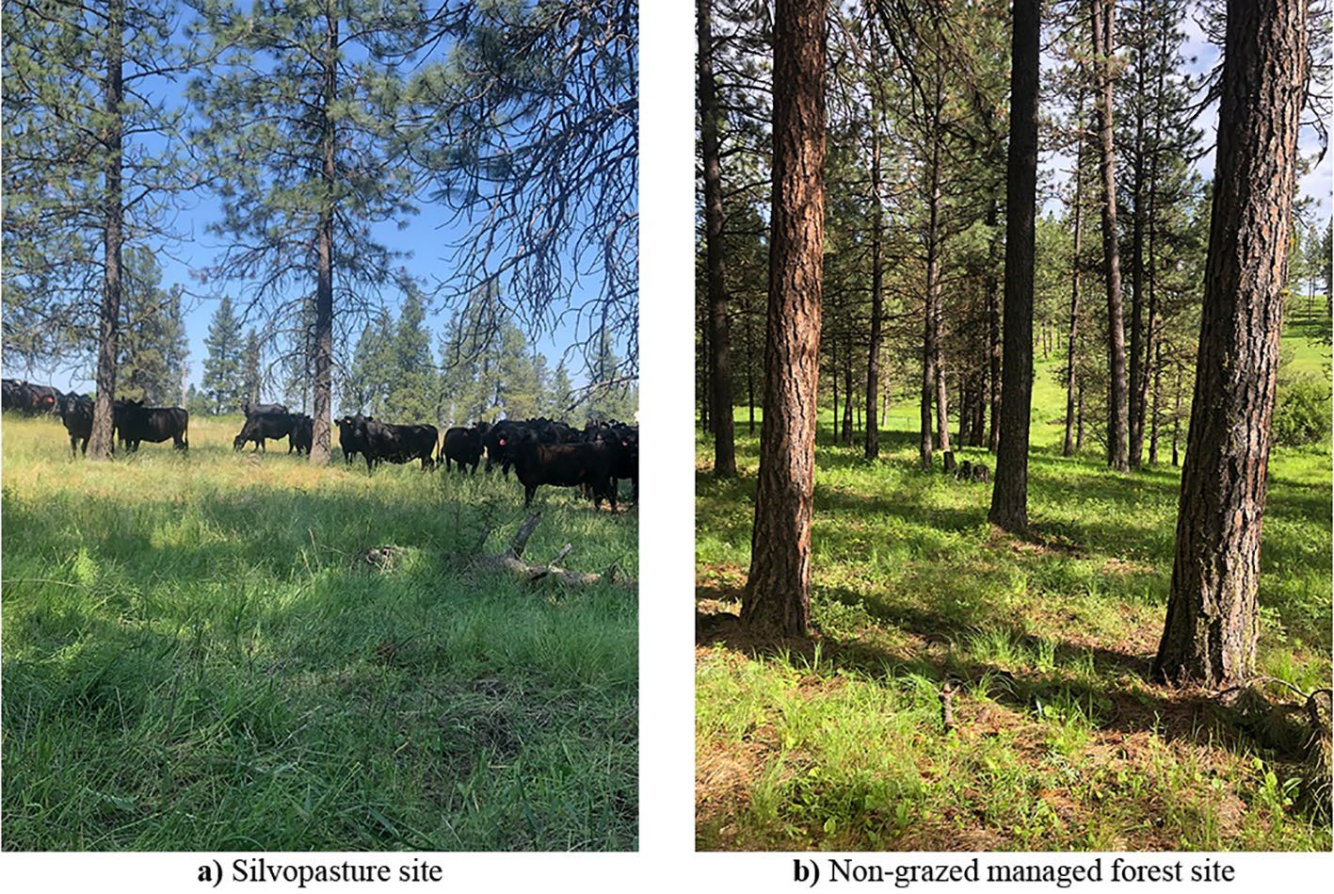
Silvopasture and non-grazed managed forest research sites in Washington, USA. (**a**) Silvopasture site with livestock grazing under *Pinus ponderosa*. (**b**) Non-grazed managed *Pinus ponderosa* forest. Photos taken by lead author.

Study sites contained two management systems: silvopasture and non-grazed managed forest (Fig. 2). Silvopasture sites have been managed for more than 10 years using rotational grazing, which is a common silvopasture management practice in this region and across the United States^26^.

Paddocks at the silvopasture sites were grazed between 15 and 20 days in a calendar year using moderate to high stocking density. To reduce variability, we selected silvopasture sites that were historically and currently grazed by cattle using a cow/calf operation (Table 2). To control grazing duration, the landowners integrated multiple paddocks into their grazing operation using portable electric fences. On both silvopasture sites, trees had been thinned to increase forage production. Tree boles were removed from the site and sold for pulp or firewood. Forage management was limited to overstory thinning and grazing. No introduction of forages had occurred in the silvopasture systems. Dominant understory plant associations include common snowberry (*Symphoricarpos albus),* Idaho fescue *(Festuca idahoensis),* and bluebunch wheatgrass *(Pseudoroegneria spicata).*

**Table 2:** Livestock composition and grazing duration at silvopasture sites.

Location	Livestock operation	Breed	Farm size (ha)	Silvopasture acreage (ha)	Average paddock size (ha)	Average herd size	Average grazing duration per paddock (days)
Cheney	Cow/calf	Angus/Aberdeen Angus cross	404	323	61	55	20
Albion	Cow/calf	Black Angus	50	37	8	20	15

Non-grazed managed forests had no history of grazing. When stand management occurred, trees were sold for timber, firewood, or pulp. Residual downed wood was pile burned. Forest structure and composition for both management systems were predominately even-aged (80–90 years) ponderosa pine (*Pinus ponderosa*) and had been thinned in the last 10 years with the goal of reducing the trees per hectare (TPH) to match historic stand structure.

For both sites, each management system contained four fixed radius plots (1/20th hectare), for a total of 16 plots in the study^37^. Plots were located 30 m inside the stand to reduce edge effects and were randomized from point of entry into the stand using a random azimuth to determine plot establishment. A GPS point was taken at plot center along with aspect and slope measurements. At each plot, we collected data on three main categories: (1) overstory measurements; (2) surface and ground fuel measurements; and (3) understory plant composition and biomass.

### Overstory measurements

From plot center, we used a Garmin GPSMAP 66s to record latitude, longitude, and elevation. Using a TruPulse 200 rangefinder, we determined the slope of the plot using the inclination function and determined site aspect using a compass. All tree species > 7 cm were measured at diameter at breast height (DBH) and were identified and measured for height and crown base height (CBH) within the fixed radius plot. Trees were also recorded as alive or dead, and the decay class of all snags was recorded^38,39^. To understand forest structure, we calculated the basal area and stand density index (SDI)^40^.

### Fuel measurements

At each plot, we established three 20 m transects to assess fuel loading using the planar intercept technique, where diameters of fuel that intercept the vertical transect are measured and converted to biomass^41,42^. Transects were established in a triangle around the plot center, such that the end of a transect marked the beginning of the second transect. Surface fuels were tallied in the first four meters of each transect using the following diameter classes: 1-h (0–0.25 inches or 0–0.6 cm), 10-h (0.25–1.0 inches or 0.6–2.5 cm), 100-h (1.0–3.0 inches or 2.5–8 cm). The diameter, species, and decay class of 1000-h fuels (> 3 inches or > 8 cm diameter) were recorded individually along each 20 m transect^38^. Duff and litter depths were measured to the nearest 0.1 cm every 2 m on each 20 m transect. If a stump or log was present at one of the sample points, we moved the point of measure 30 cm to the right of the transect and recorded litter and duff at the new sampling point. Fuel counts for 1-, 10-, and 100-h fuels were converted to mass using equations developed by Brown^41^. 1000-h fuel measurements were converted into volume using equations from Harmon and Sexton^38^. 1000-h volume estimates were converted to mass using species and decay class specific to species densities from Harmon et al.^39^ All downed woody fuels were converted to Mg ha^−1^.

### Understory plant composition and biomass

Understory plant composition was assessed using the Daubenmire method,^43^ which comprised of four 10-m transects that radiated from the plot center. Each transect was aligned in four cardinal directions. One-meter quadrats were placed at 4-m intervals along each transect (n = 2 per transect). Percent cover was estimated for annual and perennial grasses, forb and shrub species, bare mineral soil, and litter using the following cover classes: < 1, 1–5, 6–15, 16–25, 26–35, 36–45, 46–55, 56–65, 66–75, 76–85, 86–95, and > 95%. Herbaceous vegetation height was measured for each species within a quadrat and was estimated to the nearest 0.1 m. To assess biomass of understory vegetation, which was also used to assess shrub and herbaceous surface fuels, we randomly selected two quadrats along the transect. Vegetation was clipped to 2 cm above the ground level and was categorized and separated by type: graminoids (grasses, rushes and sedges), forbs, and shrubs. Vegetation was dried in an oven at 100 °C until reaching a constant weight (typically 48 h) and then weighed to the nearest 0.01 g.

## Statistical analysis

All statistical analyses were performed using R Studio V.2023.06.01^44^. A one-way ANOVA was conducted independently to compare silvopasture and non-grazed managed forest within each site. Variables analyzed included overstory structure and composition (basal area, trees per hectare, stand density index, and crown base height), surface and ground fuel loads (1-, 10-, 100-, and 1000-h fuels, litter, and duff), and understory plant composition and biomass. Differences were considered statistically significant at *p* ≤ 0.05.

## Results and discussion

### Forest overstory structure

At the northern site in Cheney, the silvopasture had significantly greater SDI, TPH, and lower CBH than the non-grazed managed forest (Table 3). In general, a greater SDI accompanied by greater TPH in this forest type typically increases surface fuels^6,45^. At the southern site in Albion, the non-grazed managed forest had significantly greater SDI and basal area than the silvopasture. We note that the mean SDI for all sites and treatments is well below the theoretical maximum for ponderosa pine (*Pinus ponderosa*) of ~ 350 based on recent research^46^. However, the non-grazed managed forest stand in Albion falls within the lower limit of self-thinning (~ 250). At this SDI, there is minimal forest production, active competition between trees for water and nutrients, and density-related mortality begins to occur^47^. To promote a more fire-resistant stand, silvopastures in this region could be managed to have spatially diverse trees by incorporating open spaces and a mosaic of clumps and individual trees. Referencing historic structure and composition of forests may help further the reduction of high severity fires^10,48^.

**Table 3:** Forest stand metrics for the silvopasture and non-grazed managed forest systems.

	Cheney			Albion		
	Silvopasture	Managed forest	*P*-value	Silvopasture	Managed forest	*P*-value
DBH (cm)	36.120 (3.906)	31.152 (1.934)	0.298	29.192 (4.468)	43.577 (11.994)	0.304
SDI	225.112 (15.332)	128.661 (18.197)	**0.007**	184.550 (19.003)	274.771 (21.228)	**0.019**
BA (m^2^)	36.770 (1.348)	32.612 (1.481)	0.083	32.605 (1.316)	51.430 (4.513)	**0.007**
CBH (m)	8.492 (0.921)	13.882 (0.587)	**0.003**	8.927 (2.776)	8.080 (2.223)	0.820
TPH	330 (37.859)	237 (40.491)	**0.016**	400 (69.761)	395 (109.048)	0.929

Values in parentheses represent standard error. *P*-values derived using a one-way ANOVA. Notation is as follows: *DBH* diameter at breast height, *SDI* stand density index, *BA* basal area, *CBH* crown base height, *TPH* trees per hectare.

Significant values are in bold.

### Fuel measurements

Litter and duff depth were significantly lower at both silvopasture sites than the non-grazed managed forests (Table 4). One contributing factor may be due to the significant reduction of grass biomass from livestock grazing at silvopasture sites when compared to non-grazed managed forest sites (Table 5). Because each silvopasture site was using rotational grazing, where the timing and duration of each grazing event was highly regulated to ensure more complete forage consumption, it likely aided in reducing litter and duff, and reduced accumulation of senesced herbaceous biomass. Ultimately, reduced litter and duff can contribute to lower intensity and rate of fire spread^34^. Reductions in litter and duff in our study sites may have also been due to the livestock themselves. A silvopasture fuel break study in France attributed reduced litter and duff to the livestock trampling and incorporating the material into the soil^34^. They also attributed reduced litter and duff to pastoral improvements, such as fertilization and planting more palatable forages that compete with shrubs^34,49^. Further evidence from Spain indicates that silvopasture systems decreases litter accumulation through trampling and manure deposition^50^.

**Table 4:** Comparison of fuel loads between silvopasture and non-grazed managed forest.

	Cheney			Albion		
	Silvopasture	Managed forest	*P*-value	Silvopasture	Managed forest	*P*-value
1-h (Mg ha^−1^)	0.052 (0.026)	0.065 (0.008)	0.667	0.062 (0.319)	0.057 (0.035)	0.920
10-h (Mg ha^−1^)	0.012 (0.006)	0.037 (0.011)	0.098	0.047 (0.017)	0.012 (0.007)	0.109
100-h (Mg ha^−1^)	0.007 (0.002)	0.010 (0.004)	0.620	0.002 (0.002)	0.005 (0.002)	0.536
1000-h (Mg ha^−1^)	4.862 (3.532)	3.072 (1.108)	0.646	0.255 (0.255)	12.635 (6.999)	0.128
Litter (cm)	1.050 (0.118)	3.650 (0.415)	**0.000**	1.940 (0.276)	3.450 (0.323)	**0.012**
Duff (cm)	0.675 (0.137)	2.432 (0.164)	**0.000**	0.657 (0.137)	1.687 (0.333)	**0.028**

Values in parentheses represent standard error. *P*-values derived using a one-way ANOVA.

Significant values are in bold.

**Table 5:** Comparison of understory vegetation types, height, and biomass between silvopasture and non-grazed managed forest.

	Cheney			Albion		
	Silvopasture	Managed forest	*P*-value	Silvopasture	Managed forest	*P*-value
Percent cover (%)						
Annual grasses	15.252 (5.399)	9.470 (2.330)	0.363	5.230 (3.711)	6.565 (4.717)	0.831
Perennial grasses	3.697 (1.444)	42.877 (6.370)	**0.001**	14.170 (5.318)	26.652 (8.465)	0.258
Shrubs	20.065 (5.282)	17.345 (6.451)	0.755	17.570 (12.222)	27.872 (8.949)	0.522
Forbs	9.010 (2.453)	17.470 (7.451)	0.322	4.857 (1.360)	12.125 (2.185)	**0.031**
Litter	94.160 (2.222)	95.782 (1.428)	0.562	92.190 (7.191)	99.687 (0.312)	0.338
Bare soil	5.535 (1.961)	4.220 (1.429)	0.608	7.190 (6.771)	0.312 (0.312)	0.349
Height (m)						
Annual grasses	0.135 (0.030)	0.400 (0.188)	0.215	0.042 (0.021)	0.090 (0.574)	0.468
Perennial grasses	0.070 (0.031)	0.340 (0.026)	**0.001**	0.145 (0.015)	0.390 (0.036)	**0.001**
Shrubs	0.232 (0.048)	0.342 (0.035)	0.116	0.220 (0.049)	0.395 (0.082)	0.118
Forbs	0.145 (0.005)	0.185 (0.008)	**0.007**	0.150 (0.014)	0.147 (0.007)	0.885
Biomass (g/m^2^)						
Grasses	18.927 (4.139)	69.940 (8.700)	**0.002**	18.270 (2.946)	58.262 (11.992)	**0.017**
Forbs	17.245 (1.257)	23.245 (6.810)	0.420	15.087 (7.080)	14.465 (2.382)	0.936
Shrubs	13.125 (1.766)	24.515 (7.622)	0.202	37.355 (17.892)	31.757 (18.940)	0.920

Values in parentheses represent standard error. *P*-values derived using a one-way ANOVA.

Significant values are in bold.

Reducing litter and duff has important implications for fire management. There is a correlation between rate of fire spread and burn severity with depth of litter and duff, particularly in this forest type^51^. Forests where fire suppression has occurred may have high duff loads, which may burn for long periods of time as a smoldering fuel. In some cases, smoldering duff can result in prolonged heat loading, which can increase tree root mortality and increased stress to trees^52^. By reducing litter and duff where fire suppression has occurred, fire-related mortality or tree stress may be reduced when a fire does occur^53^.

There was no statistical significance among the 1-, 10-, 100-, and 1000-h fuels between silvopasture and non-grazed managed forests despite variability in stand density and TPH (Table 4). The lack of significant variability in fuel loads between management systems is attributed to forest management objectives and long-term fuels management. On our study sites, tree boles were removed and primarily sold for timber, firewood, or wood pulp. Further, residual woody biomass that was not sold was pile burned or used for personal use on both the silvopasture and managed forest sites. This method of whole tree harvest is known to be an effective management strategy for reducing surface fuels^6^. Producers in our study were also removing residual woody debris in their silvopastures to increase the surface area for forages to grow. The act of removing residual woody debris in silvopastures increases forage production and likely doubles as a fuels management treatment. As such, the degree to which silvopasture impacts these fuel types is likely producer-dependent and related to how clean they want to maintain the understory for forages to grow.

### Forest understory and shrub and herbaceous biomass

Percent ground covered by perennial grasses were significantly lower in the Albion silvopasture site than non-grazed managed forest site (Table 5). This finding is not surprising given that the perennial grasses are the more preferred forages by livestock on these sites, particularly in the summer^17^. Further, there was a significant difference in perennial grass species composition between management systems. At the southern site, the silvopasture system was predominantly composed of *Arrhenatherum elatus* and *Festuca idahoensis* while the non-grazed managed forest was primarily composed of *Arrhenatherum elatus* and *Bromus inermis*. At the northern site, the silvopasture was primarily composed of *Festuca idahoensis*, *Elymus repens*, and *Poa* species while the non-grazed managed forest was primarily composed of *Festuca idahoensis* and *Phalaris arundicancea*. Given similar site characteristics between management systems, differences between perennial grass species are likely driven by livestock grazing. While these perennial grasses contribute to surface fuels, producers and fires managers in the region are primarily concerned with invasive annual grasses, since they tend to senesce earlier and increase fuel accumulation and thus alter fire regimes^54^.

Percent cover of annual grasses, shrubs, bare mineral soil, and litter did not differ between management systems (Table 5). However, differences in vegetation height were noted for perennial grasses, which were shorter at silvopasture sites than non-grazed forests sites. We attribute these differences to grazing pressure. Significant differences in total biomass between understory vegetation type was also noted for grasses, with silvopasture having lower biomass than non-grazed managed forest sites. Shrub and forb biomass were similar between management systems.

The change in species composition, biomass, and height in this study was likely driven by livestock grazing. These results are supported by prior research showing that the intensity and duration at which cattle are grazed can alter species composition^4,15^, and reduce biomass from certain fuel classes potentially leading to reduced fire severity and intensity^25,55^. More deliberate management of forages through nutrient additions, seeding of more palatable species, and or grazing management could further alter species composition. For example, researchers found that seeding of more palatable forages in a silvopasture resulted in more complete forage consumption and thus reduced fuel loads^35^. However, this strategy could have the opposite effect and increase fuel loads if grazing pressure cannot match increases in plant growth^49^, illustrating the importance of carefully considering all the system components when managing a silvopasture.

## Limitations of the study

The interpretation and application of results from this study is limited to the climate and forest type in which this study occurred. This study is also limited by the number of sites, which were difficult to find for several reasons. The first is that many producers in the region do not refer to their management system as silvopasture and instead refer to it as woodland grazing^26^. This made identifying potential producers for study sites difficult, as copious time and effort was utilized to conduct interviews to discern forest, forage, and livestock management methods. Further, when silvopasture sites were identified, few properties had ungrazed forest which was necessary for comparative analysis. Ideally, this study would have included treatments comparing silvopasture to a matrix of forest management and grazing intensities to determine outcomes of each management system. This issue points to the need for long term research sites to be established on working silvopastures to determine mechanistic effects of various forest and grazing management combinations on the same site.

## Implications and future research

As climate change and land management continue to alter forest structure, managing wildfires has become increasingly difficult, especially with the expansion of the wildland urban interface. Research on additional management strategies that reduce fuel loads and maintain desired ecological characteristics of forests may provide beneficial insights. In designing management strategies, it will be important to account for the ecological and economic objectives of land managers and ensure benefits received now do not exacerbate problems in the future.

Our research offers evidence that silvopasture reduces select fuel types and select vegetative biomass when compared to managed forests of the Interior Columbia Basin. However, 1-, 10-, 100-, and 1000-h fuels were similar between the management systems. Based on the stand density index and TPH of the two silvopastures, they may benefit from a further reduction in trees per hectare to improve forage production. Thinning at these sites could also create more spatial heterogeneity and mimic historic stand structure, further reducing potential fire severity. This may be important given the increase in fire risk due to climate change. However, producers using silvopasture in this region must balance fewer trees per hectare, which can improve forage production and thus livestock stocking, with the economic implications associated with a reduction in timber sales. It is likely that this balance of forest management, forage production, and livestock stocking, is producer-dependent based on their goals and localized markets.

To date, the scientific literature focused on the impact of silvopasture management on forest structure and herbaceous fuel loads is limited. Research showing the impact of varying stand density and canopy cover on fuel loads and understory biomass would be beneficial, as this would help inform producers of the optimal balance between tree density, forage production, and livestock stocking that match site capability and their desired management goals. Future research characterizing the impact silvopasture management has on fuel loads in other forest types and regions across the world would be beneficial, as most of the research is exclusive to the Mediterranean region. Finally, long term silvopasture research sites would be beneficial to investigate how continued use of silvopasture management effects the ecological trajectory of a site and the associated changes in fuel load composition through time.

## Data Availability

Please contact the lead author, Mark Batcheler, to request data from this study.
